# Optimal Recovery Following Pediatric Concussion

**DOI:** 10.1001/jamanetworkopen.2025.1092

**Published:** 2025-03-19

**Authors:** Miriam H. Beauchamp, Ken Tang, Andrée-Anne Ledoux, Ashley D. Harris, Kristina A. Kowalski, William R. Craig, Jocelyn Gravel, Quynh Doan, Stephen B. Freedman, Roger L. Zemek, Keith Owen Yeates

**Affiliations:** 1 Department of Psychology, University of Montreal, Montreal, Quebec, Canada; 2 Ste-Justine Hospital Azrieli Research Center, Montreal, Quebec, Canada; 3 Independent statistical consultant, Richmond, British Columbia, Canada; 4 Children’s Hospital of Eastern Ontario Research Institute, Ottawa, Ontario, Canada; 5 School of Psychology, Faculty of Social Sciences, University of Ottawa, Ottawa, Ontario, Canada; 6 Department of Cellular and Molecular Medicine, Faculty of Medicine, University of Ottawa, Ottawa, Ontario, Canada; 7 Department of Radiology, University of Calgary, Calgary, Alberta, Canada; 8 Alberta Children’s Hospital Research Institute, Calgary, Alberta, Canada; 9 Department of Psychology, University of Calgary, Calgary, Alberta, Canada; 10 Department of Pediatrics, University of Alberta and Stollery Children’s Hospital, Edmonton, Alberta, Canada; 11 Department of Pediatric Emergency Medicine, CHU Sainte-Justine, Université de Montréal, Montreal, Quebec, Canada; 12 Department of Pediatrics, University of British Columbia, Vancouver, British Columbia, Canada; 13 BC Children’s Hospital Research Institute, Vancouver, British Columbia, Canada; 14 Department of Pediatrics, Cumming School of Medicine, University of Calgary, Calgary, Alberta, Canada; 15 Department of Emergency Medicine, Cumming School of Medicine, University of Calgary, Calgary, Alberta, Canada; 16 Department of Pediatrics and Emergency Medicine, University of Ottawa, Ottawa, Ontario, Canada; 17 Alberta Children’s Hospital Research Institute, University of Calgary, Calgary, Alberta, Canada; 18 Hotchkiss Brain Institute, University of Calgary, Calgary, Alberta, Canada

## Abstract

**Importance:**

Pediatric concussion affects millions and results in heterogeneous outcomes and recovery trajectories. Given favorable outcome for most children, it is useful to understand characteristics of positive outcome to promote full recovery in all children.

**Objective:**

To document the timeframe of recovery to optimal functioning, defined comprehensively across motor-physical, cognitive, socioemotional, and resilience-support domains, after concussion among children ages 8 to 16 years.

**Design, Setting, and Participants:**

For this prospective cohort study, children ages 8 to 16.99 years with a concussion or orthopedic injury (OI) were recruited between September 2016 and July 2019 from 5 Pediatric Emergency Research Canada emergency departments and assessed approximately 10 days, 3 months, and 6 months after their injury. Data were analyzed from January 29, 2024, to January 11, 2025.

**Exposure:**

Concussion.

**Main Outcomes and Measures:**

Participants completed self-report and direct assessment measures of postconcussive symptoms, physical activity and function, balance, cognitive function, quality of life, resilience, and social support. The main outcome was optimal functioning, which was derived from 11 variables and criteria indicative of absence of impairment and average or above functioning in each domain (overall score, 0-11; higher score indicates better function). A longitudinal, multivariable, cumulative probability ordinal regression model was fitted to examine factors associated with optimal functioning.

**Results:**

A total of 967 children (median [IQR] age, 12.3 [10.5-14.3] years; 562 [58.1%] male) were enrolled, including 633 children with a concussion and 334 children with an OI. The median (IQR) optimal functioning scores for the OI group were 6.0 (4.0-8.0) at 10 days, 7.0 (5.0-9.0) at 3 months, and 7 (5.0-9.0) at 6 months, compared with 4.0 (2.0-6.0) at 10 days, 6.0 (4.0-9.0) at 3 months, and 7.0 (4.0-9.0) at 6 months in the concussion group. The 3 main variables (time, sex, and group) were significantly associated with optimal functioning, as were all 2-way interactions. Time was the strongest factor associated with optimal functioning (Wald χ^2^_58_ = 485.11; *P* < .001), followed by group (Wald χ^2^_6_ = 95.10; *P* < .001), and sex (Wald χ^2^_6_ = 23.19; *P* < .001). At the 10-day follow-up, concussion was associated with lower optimal functioning than OI among females (odds ratio [OR],  0.24 [95% CI, 0.16-0.36]) and males (OR, 0.37 [95% CI, 0.26-0.53]). This difference persisted for females at 3 months (OR, 0.57 [95% CI, 0.35-0.93]) but not for males. Optimal functioning was comparable at 6 months.

**Conclusions and Relevance:**

In this prospective cohort study of children with concussion, achieving optimal functioning levels across physical, cognitive, socioemotional, and resilience domains took 3 months or more, especially for girls with concussion. Multiple domains of outcome need to be taken into account when considering full recovery and optimal function after pediatric concussion.

## Key Points

**Question:**

How does optimal functioning evolve after pediatric concussion compared with orthopedic injury and what factors are associated with recovery?

**Findings:**

In this cohort study of 967 children ages 8 to 16 years, including 633 children with concussion and 334 children with orthopedic injury, children with concussion had lower optimal functioning and females were less likely to show optimal functioning than males up to 6 months after injury.

**Meaning:**

These findings suggest that attaining optimal functioning may take 3 months or more after pediatric concussion when multiple domains of outcome are taken into account, especially for girls.

## Teaser

This cohort study examines the timeframe for recovery among children ages 8 to 16 years with concussion compared with children with orthopedic injury.

## Introduction

Concussion affects millions of children annually, leading to physical, cognitive, sleep, and emotional symptoms that typically resolve within 4 weeks of injury.^1^ Approximately 30% of children experience persisting symptoms beyond that time, which can impact academic and psychological functioning and reduce quality of life.^2,3,4,5,6^ Given most children return to preinjury levels of function within a few weeks, there is a need to better understand how recovery optimally evolves and what distinguishes children who recover well from those who do not.

Optimal functioning is poorly defined, and little is known of what contributes to good recovery. To date, work has mostly focused on how individual predispositions, such as psychological resilience, affect outcome.^7,8,9,10,11^ Adopting a comprehensive view of optimal outcome, Beauchamp and colleagues^12^ studied the concept through the lens of wellness, defined as a state of complete physical, mental, and social well-being, and not merely the absence of deficits. They operationalized this concept by combining common outcome indicators such that children who sustained concussion were characterized as being well if they had no persisting symptoms, no cognitive difficulties, and above average quality of life. Using this approach, 41.5% of participants in the study by Beauchamp and colleagues^12^ met the wellness criteria 4 weeks after concussion, increasing to 52.2% by 12 weeks after injury. Children who were younger, sustained concussion in sports or recreation settings, did not have preinjury developmental problems, and had better acute working memory were more likely to show optimal recovery. Using a similar, comprehensive approach to define optimal outcome in a cohort of children who sustained concussion during early childhood (18-60 months), Aubuchon and colleagues^13^ found that fewer children with concussion had optimal functioning over 6 and 18 months after injury compared with children with orthopedic injuries or typical development.

Pediatric concussion prognosis and outcome are heterogeneous,^1^ and the best models of optimal functioning and well-being are likely to consider a broad range of domains. In the study by Beauchamp et al,^12^ no comparison group was available to examine whether the reduction in wellness was associated with brain injury or with traumatic injury more generally. The specific aims of this study were to examine the proportion of children with optimal outcome across motor-physical, cognitive, socioemotional, and resilience-support domains compared with children with orthopedic injury (OI); document the evolution of optimal outcome over time (10 days, 1 month, 3 months after their injury); and explore whether sex moderates optimal functioning. We expected that children with concussion would have fewer indicators of optimal functioning than those with OI, especially 10 days after injury, that optimal functioning would increase over time, and that group differences would be more pronounced among females.

## Methods

This cohort study was approved by local research ethics committees at each site. All participants provided assent, with written consent provided by parents or guardians. Reporting follows the Strengthening the Reporting of Observational Studies in Epidemiology (STROBE) reporting guideline.

### Design and Setting

This is a secondary analysis of a prospective, longitudinal cohort, the Advancing Concussion Assessment in Pediatrics (A-CAP)^14^ study. Recruitment occurred from September 2016 to July 2019 during acute emergency department visits at 5 Pediatric Emergency Research Canada network sites: Alberta Children’s Hospital (Calgary), Children’s Hospital of Eastern Ontario (Ottawa), Ste-Justine Hospital (Montreal), Stollery Children’s Hospital (Edmonton), and British Columbia Children’s Hospital (Vancouver).

### Participants

Children between ages 8.00 and 16.99 years were recruited less than 48 hours after injury. Children in the concussion group sustained a blunt head trauma resulting in at least 1 of the following criteria, consistent with the World Health Organization mild traumatic brain injury definition: observed loss of consciousness (LOC), or Glasgow Coma Scale (GCS) 13 to 14, or at least 1 acute sign or symptom of concussion (ie, posttraumatic amnesia, focal neurological deficits, skull fracture, posttraumatic seizure, vomiting, headache, dizziness, other mental status changes). Children were excluded if they demonstrated neurologic deterioration (GCS <13), required neurosurgery, had LOC more than 30 minutes or posttraumatic amnesia more than 24 hours. Children in the OI group were recruited to control for common injury risk factors and to account for the general effects of traumatic injury. They sustained an upper or lower extremity fracture, sprain or strain, secondary to blunt force trauma, associated with an abbreviated injury scale score of 4 or less. Exclusion criteria for the OI group were head trauma or signs or symptoms of concussion at recruitment, or any injury requiring surgery or procedural sedation. Exclusion criteria applicable to both groups were any injury with an abbreviated injury scale score more than 4; hypoxia, hypotension, or shock during or after the injury; previous concussion within 3 months or any traumatic brain injury requiring hospitalization; preinjury neurologic disorder; severe neurodevelopmental disability; trauma as a result of violence or self-harm; or severe psychiatric disorder requiring hospitalization within the past year.

### Procedures

Detailed procedures are in the published protocol.^14^ Briefly, at the time of recruitment, research staff collected demographic and acute injury information and assessed balance and neurocognitive function. Participants were then invited to complete in-person assessments at approximately 10 days (postacute), 3 months, and 6 months after injury. Demographic information included age, sex, race and ethnicity, parental education, and material and social deprivation indices. Race and ethnicity were reported by parents or caregivers and classified as Asian, Black, Hispanic, Indigenous, White, and other or multiracial (an original survey option that respondents could self-select). Race and ethnicity were collected to provide information on the representativeness of the participant sample.

### Measures

Direct (child assessment) and indirect (child self-report questionnaires) measures were completed at the 3 time points. Parent ratings of preinjury postconcussive symptoms (PCS) were collected retrospectively. All measures are validated and reliable.^14,15,16,17,18,19,20,21^ Brief descriptions of the measures are provided in Table 1.

**Table 1.** Measures and Criteria Used to Define Optimal Functioning

Measure	Brief description	Variable used	Criterion for optimal functioning
**Motor-physical**				
Healthy lifestyle behaviors questionnaire^15^	26 Items on frequency of mental and physical activity (including rest), sleep and screen time	Total weekly physical activity	Participants had to meet the Canadian Government recommendation of 60 min of physical activity per day (420 min or more per week)^21^
HBI^14^	20-Item child self-report of PCS frequency	Physical-somatic PCS (9 items)	Cutoff based on the OI sample median at the 10-day time point
Balance error scoring system^16^	Participants are asked to balance with hands on hips and eyes closed for 20 s; error points are given for opening eyes, lifting hands off hips or stepping, stumbling, or falling	Tandem Stance Total Error score (maximum 10)	≤3 Errors on tandem stance^6^
PedsQL^17^	Ratings of health-related QOL	Physical Functioning score (8 items)	Score on physical subscale at or above the population mean; age appropriate norms for ages 6-7, 8-12, 13-18 y^17^
**Cognitive**				
Central nervous system vital signs^18^	Computerized cognitive test battery; 3 of the subtest scores were used as a an indicator of cognitive processing speed	Composite score from the reaction time, cognitive flexibility and visual memory subtests	Participants had to have average or above performance based on standardized norms from 1600 individuals age 8-90 y
HBI^14^	10-Item child self-report of PCS frequency	Cognitive PCS (11 items)	The cutoff was based on the OI sample median at the 10-day time point
PedsQL	Ratings of health-related QOL	School Functioning score (5 items)	Score on school subscale at or above the population mean; age appropriate norms for ages 6-7, 8-12, 13-18 y^17^
**Socioemotional**				
PedsQL	Ratings of health-related QOL	Social Functioning score (5 items)	Score on social subscale at or above the population mean; age appropriate norms for ages 6-7, 8-12, 13-18 y^17^
PedsQL	Ratings of health-related QOL	Emotional Functioning score (5 items)	Score on emotional subscale at or above the population mean; age appropriate norms for ages 6-7, 8-12, 13-18 y^17^
**Resilience-support**				
Connor-Davidson Resilience scale^19^	10-Item child rating of perceived psychological resilience on a 5-point Likert scale	Total score	The cutoff was based on the OI sample median at the 10-day time point
Child and Adolescent Social Support scale^20^	40-Item scale of perceived social support received from parents, teachers, classmates, and friends on a 6-point Likert scale	Sum of scores from 4 sources (40 points)	The cutoff was based on the OI sample median at the 10-day time point

Abbreviations: HBI, Health and Behavior Inventory; OI, orthopedic injury; PCS, postconcussive symptom; PedsQL, pediatric quality of life inventory; QOL, quality of life.

### Definition of Optimal Outcome

The criteria used to define optimal outcome were similar to those used by Beauchamp et al,^12^ including PCS, cognition, and quality of life, but capitalized on a fuller set of measures for a more comprehensive approach. Four domains of functioning (motor-physical, cognitive, socioemotional, resilience-support) were targeted, each including 2 to 4 variables, at least 1 of which was a positive indicator. Table 1 presents the variables for each measure and domain and criteria used for optimal outcome classifications. When published guidelines, norms, or standardized cutoffs were available, they were used to define optimal thresholds. Otherwise, cutoffs were proposed based on the OI sample median score at the postacute time point. Participants were considered to have optimal functioning if they met all criteria for each variable in each domain.

### Derivation of the Optimal Functioning Score and Subscores

To derive an optimal functioning score, the number of optimal functioning measures available for each participant was initially assessed. If a participant had at least 8 of 11 measures (>70%), then completeness was considered satisfactory, and a tally of the number of measures that met the optimal functioning criterion (proposed score cutoff) was computed (range, 0-11, with 11 indicating maximal optimal functioning), with incomplete measures assumed as 0. If completeness was less than 70%, no score was derived. Similarly, for subscores, if a participant completed all constituent measures for the domain (ie, no missing measures allowed), then a tally of the number of measures that met the associated criterion was computed. The maximum possible subscores are motor-physical, 4; cognitive, 3; socioemotional, 2; and support-resilience, 2.

### Statistical Analysis

All analyses were performed using *R* software version 4.2.2 (R Project for Statistical Computing). Statistical modeling was conducted with functions from the *rms* package. Descriptive statistics (median, frequency, and percentage) were used to summarize participant characteristics (sociodemographic attributes, medical history, injury mechanism, and preinjury cognitive and somatic PCS) and the 11 optimal functioning variables and subscores stratified by group. At each time point, median, IQR, mean, SD, minimum, maximum, and the number and percentage of participants who met the cutoff criterion for each measure are presented.

To examine the associations of 3 main variables (group, time, sex) with optimal functioning, a longitudinal, multivariable, cumulative probability ordinal regression model was fitted for the optimal functioning score, with cluster-adjusted SEs to correct for repeated measurement.^22^ The model specification featured a 3-way interaction among group, time, and sex, plus a comprehensive set of 10 demographic and preinjury information covariates, including age, race and ethnicity, parental education, material deprivation index, social deprivation index, concussion history, migraine history, injury mechanism, and preinjury cognitive and somatic PCS scores. Interactions with time were also specified for all 10 covariates. To relax the linearity assumption and allow for a more flexible association with the optimal functioning score, restricted cubic splines with 3 knots (ie, pivot points) were applied to all continuous covariates. Removal of any covariates was not considered after initial model specification. A complete-case analysis approach was applied, as listwise deletion was performed on observations with missing values. To evaluate the correlation between individual factors, covariates, and outcome, an overall Wald χ^2^ test for each factor and covariate (inclusive of any associated nonlinear/interaction components) was assessed. To further detail the magnitude and directionality of the estimated covariate-adjusted associations of the main factors, partial effect plots illustrate the predicted median of optimal functioning by group, sex, and time, while all other model covariates are held constant at their median (for continuous variables) or mode (for categorical variables). Postmodel fit contrasts were performed to quantify effect sizes. Estimated associations are quantified in terms of common odds ratios (ORs) and associated 95% CIs. ORs derived from the cumulative probability ordinal regression models represent the estimated odds of having a higher optimal functioning score for individuals in the exposure group (eg, history of migraines) compared with the reference group (eg, no history of migraines). CIs provide a range of possible values that the true population OR lies within a specified degree of confidence (eg, 95%). *P* values were 2-sided, and statistical significance was set at *P* ≤ .05. Data were analyzed from January 29, 2024, to January 11, 2025.

## Results

### Participant Characteristics

Among 967 (median [IQR] age, 12.3 [10.5-14.3] years; 562 [58.1%] male) consented study participants, 633 (65.5%) had concussion and 334 (34.5%) had OI. Few participants (175 [18.5%]) had a previous concussion with maximum symptom duration of at least 1 week, and 57 participants (6.0%) reported a history of migraine. Full sample details are provided in Table 2.

**Table 2.** Characteristics of Eligible Study Participants by Group

Variable	Participants, No. (%)			
	OI (n = 334)	Concussion (n = 633)	Total (n = 967)
Site			
Calgary	76 (22.8)	150 (23.7)	226 (23.4)
Edmonton	64 (19.2)	120 (19.0)	184 (19.0)
Montreal	29 (8.7)	71 (11.2)	100 (10.3)
Ottawa	56 (16.8)	161 (25.4)	217 (22.4)
Vancouver	109 (32.6)	131 (20.7)	240 (24.8)
Age, median (IQR), y	12.5 (10.9-14.3)	12.0 (10.2-14.4)	12.3 (10.5-14.3)
Sex			
Female	151 (45.2)	254 (40.1)	405 (41.9)
Male	183 (54.8)	379 (59.9)	562 (58.1)
Race and ethnicity (n = 834)^a^			
Asian	23 (8.2)	52 (9.4)	75 (9.0)
Black	7 (2.5)	21 (3.8)	28 (3.4)
Hispanic	12 (4.3)	13 (2.3)	25 (3.0)
Indigenous	3 (1.1)	13 (2.3)	16 (1.9)
White	197 (70.4)	386 (69.7)	583 (69.9)
Other or multiracial	38 (13.6)	69 (12.5)	107 (12.8)
Parental education (n = 824)			
≤High school	43 (15.6)	86 (15.7)	129 (15.7)
Trades or 2-year college	83 (30.2)	164 (29.9)	247 (30.0)
Bachelor’s degree	93 (33.8)	206 (37.5)	299 (36.3)
>Bachelor’s degree	56 (20.4)	93 (16.9)	149 (18.1)
Material Deprivation Index, median (IQR), percentile (n = 918)	27.0 (12.0-56.0)	28.0 (11.0-54.5)	28.0 (12.0-55.0)
Social Deprivation Index, median (IQR), percentile (n = 918)	43.0 (23.5-66.5)	41.0 (23.0-66.0)	42.0 (23.0-66.0)
Mechanism of injury (n = 867)			
Bicycle-related	12 (4.2)	9 (1.5)	21 (2.4)
Fall	157 (54.9)	267 (46.0)	424 (48.9)
Motor vehicle collision	0	10 (1.7)	10 (1.2)
Struck object	63 (22.0)	184 (31.7)	247 (28.5)
Struck person	33 (11.5)	105 (18.1)	138 (15.9)
Other	21 (7.3)	6 (1.0)	27 (3.1)
Previous concussion maximum symptom duration, No. (%) (n = 947)			
<1 wk or no previous concussions	273 (83.5)	499 (80.5)	772 (81.5)
≥1 wk	54 (16.5)	121 (19.5)	175 (18.5)
Child history of migraine (n = 945)	23 (7.0)	34 (5.5)	57 (6.0)
HBI cognitive retrospective preinjury, median (IQR) (n = 836)	6.0 (0.0-12.0)	8.0 (2.0-15.0)	7.0 (1.0-14.0)
HBI somatic retrospective preinjury, median (IQR) (n = 836)	1.0 (0.0-3.0)	2.0 (0.0-4.0)	1.0 (0.0-4.0)

^a^ Race and ethnicity were reported by the participant’s primary caregiver on an in-house sociodemographic questionnaire. The other or multiracial category was an original survey option that respondents could self-select.

### Optimal Functioning Specific Components

All 11 measures were reasonably well completed among study participants; with available data ranging from 688 participants (71.1%) to 829 participants (85.7%) across time points. eTable 1 in Supplement 1 presents the summary statistics and group differences on individual components of the optimal functioning score. Overall, the percentage of participants meeting specific optimal functioning criteria (score cutoff) tended to increase across time.

### Optimal Functioning Score and Domain Subscores

The overall optimal functioning score was computed for 824 participants (85.2%) at 10 days, 725 participants (75.0%) at 3 months, and 697 participants (72.0%) at 6 months, after considering completeness requirements (Table 3). For the OI group, the median (IQR) score was 6.0 (4.0-8.0) at 10 days, 7.0 (5.0-9.0) at 3 months, and 7.0 (5.0-9.0) by 6 months. For the concussion group, the median (IQR) score was 4.0 (2.0-6.0) at 10 days, 6.0 (4.0-9.0) at 3 months, and 7.0 (4.0-9.0) by 6 months. Optimal functioning score distributions over time by group are presented in the eFigure in Supplement 1.

**Table 3.** Summary Statistics for the Optimal Functioning Score and Subscores

Score/domain	Sample, No.	OI (n = 334)		Concussion (n = 633)		Total (n = 967)	
		Median (IQR)	Mean (SD) [range]	Median (IQR)	Mean (SD) [range]	Median (IQR)	Mean (SD) [range]
Optimal Functioning score							
10 d	824	6.0 (4.0-8.0)	6.0 (2.5) [1.0-11.0]	4.0 (2.0-6.0)	4.1 (2.4) [0.0-11.0]	5.0 (3.0-7.0)	4.8 (2.6) [0.0-11.0]
3 mo	725	7.0 (5.0-9.0)	7.0 (2.7) [0.0-11.0]	6.0 (4.0-9.0)	6.3 (2.9) [0.0-11.0]	7.0 (4.0-9.0)	6.5 (2.8) [0.0-11.0]
6 mo	697	7.0 (5.0-9.0)	7.0 (2.8) [1.0-11.0]	7.0 (4.0-9.0)	6.6 (2.9) [0.0-11.0]	7.0 (4.0-9.0)	6.7 (2.8) [0.0-11.0]
Motor-physical							
10 d	763	2.0 (2.0-3.0)	2.4 (1.0) [0.0-4.0]	1.0 (1.0-2.0)	1.5 (1.0) [0.0-4.0]	2.0 (1.0-2.0)	1.8 (1.1) [0.0-4.0]
3 mo	700	3.0 (2.0-4.0)	2.8 (1.1) [0.0-4.0]	3.0 (2.0-4.0)	2.6 (1.1) [0.0-4.0]	3.0 (2.0-4.0)	2.7 (1.1) [0.0-4.0]
6 mo	680	3.0 (2.0-4.0)	2.8 (1.0) [0.0-4.0]	3.0 (2.0-4.0)	2.8 (1.1) [0.0-4.0]	3.0 (2.0-4.0)	2.8 (1.1) [0.0-4.0]
Cognitive							
10 d	811	2.0 (1.0-2.0)	1.6 (0.9) [0.0-3.0]	1.0 (0.0-1.0)	1.0 (0.9) [0.0-3.0]	1.0 (0.0-2.0)	1.2 (0.9) [0.0-3.0]
3 mo	701	2.0 (1.0-3.0)	1.8 (1.0) [0.0-3.0]	2.0 (1.0-2.0)	1.6 (1.0) [0.0-3.0]	2.0 (1.0-3.0)	1.7 (1.0) [0.0-3.0]
6 mo	685	2.0 (1.0-3.0)	1.8 (1.0) [0.0-3.0]	2.0 (1.0-3.0)	1.7 (1.0) [0.0-3.0]	2.0 (1.0-3.0)	1.7 (1.0) [0.0-3.0]
Socioemotional							
10 d	828	1.0 (1.0-2.0)	1.2 (0.8) [0.0-2.0]	1.0 (0.0-2.0)	0.9 (0.8) [0.0-2.0]	1.0 (0.0-2.0)	1.0 (0.8) [0.0-2.0]
3 mo	727	1.0 (1.0-2.0)	1.3 (0.8) [0.0-2.0]	1.0 (1.0-2.0)	1.2 (0.8) [0.0-2.0]	1.0 (1.0-2.0)	1.2 (0.8) [0.0-2.0]
6 mo	699	1.0 (1.0-2.0)	1.2 (0.8) [0.0-2.0]	1.0 (0.0-2.0)	1.2 (0.8) [0.0-2.0]	1.0 (1.0-2.0)	1.2 (0.8) [0.0-2.0]
Resilience-support							
10 d	820	1.0 (0.0-2.0)	1.0 (0.8) [0.0-2.0]	1.0 (0.0-1.0)	0.8 (0.7) [0.0-2.0]	1.0 (0.0-1.0)	0.9 (0.8) [0.0-2.0]
3 mo	724	1.0 (0.0-2.0)	1.0 (0.8) [0.0-2.0]	1.0 (0.0-2.0)	1.0 (0.8) [0.0-2.0]	1.0 (0.0-2.0)	1.0 (0.8) [0.0-2.0]
6 mo	695	1.0 (0.2-2.0)	1.1 (0.8) [0.0-2.0]	1.0 (0.0-2.0)	1.0 (0.8) [0.0-2.0]	1.0 (0.0-2.0)	1.0 (0.8) [0.0-2.0]

Optimal functioning subscores were computed for 680 to 828 participants (70.3%-85.6% of the eligible sample) across the 4 domains and 3 study time points by group (Table 3). Overall, the motor-physical and cognitive subscore medians increased between the 10-day and 6-month follow-ups, but the socioemotional and resilience-support subscores remained constant. Both the medians and means of all 4 optimal functioning subscores in the concussion group were either lower than or equal to those in the OI group across all time points.

### Associations Among Group, Time, and Sex With Optimal Functioning

eTable 2 in Supplement 1 presents a comparison of baseline characteristics between participants included (at least 1 of the 3 study time points had complete data) and excluded from the final statistical model. The multivariable model provided evidence of an association among the 3 main factors (group, time, sex) with optimal functioning (Table 4). All 2-way interactions among the 3 variables were significant, with group × time being especially strong (Wald χ^2^_4_ = 62.49; *P* < .001). Time was the strongest factor (Wald χ^2^_58_ = 485.11; *P* < .001), followed by group (Wald χ^2^_6_ = 95.10; *P* < .001), and then sex (Wald χ^2^_6_ = 23.19; *P* < .001). The 3-way interaction among group, sex, and time did not reach statistical significance (Wald χ^2^_2_ = 2.62; *P* = .27).

**Table 4.** Wald χ^2^ From Multivariable Model Fit Examining Factors Associated With Optimal Functioning^a^

Factor	χ^2^ (*df*)	*P* value
Time		
Factor + higher order factors	485.11 (58)	<.001
All interactions	184.60 (56)	<.001
Group		
Factor + higher order factors	95.10 (6)	<.001
All interactions	65.78 (5)	<.001
Sex		
Factor + higher order factors	23.19 (6)	<.001
All interactions	18.45 (5)	.002
Age		
Factor + higher order factors	14.86 (6)	.02
All interactions	9.03 (4)	.06
Nonlinear (factor + higher order factors)	9.70 (3)	.02
Race		
Factor + higher order factors	27.17 (15)	.03
All interactions	16.98 (10)	.08
Parental education		
Factor + higher order factors	19.07 (9)	.03
All interactions	10.02 (6)	.12
Material Deprivation Index		
Factor + higher order factors, percentile	12.95 (6)	.04
All interactions	9.45 (4)	.05
Nonlinear (factor + higher order factors)	5.96 (3)	.11
Social Deprivation Index		
Factor + higher order factors, percentile	3.50 (6)	.74
All interactions	2.58 (4)	.63
Nonlinear (factor + higher order factors)	1.44 (3)	.70
Mechanism of injury		
Factor + higher order factors	32.50 (15)	.006
All interactions	15.84 (10)	.10
Previous concussion maximum symptom duration		
Factor + higher order factors	5.28 (3)	.15
All interactions	4.11 (2)	.13
Child history of migraine		
Factor + higher order factors	9.76 (3)	.02
All interactions	8.18 (2)	.02
HBI cognitive retrospective pre-injury		
Factor + higher order factors	61.73 (6)	<.001
All interactions	10.36 (4)	.04
Nonlinear (factor + higher order factors)	5.43 (3)	.14
HBI somatic retrospective pre-injury		
Factor + higher order factors	27.35 (6)	<.001
All interactions	5.00 (4)	.29
Nonlinear (factor + higher order factors)	6.78 (3)	.08
**Specific interactions**		
Group × sex (factor + higher order factors)	6.22 (3)	.10
Time × group (factor + higher order factors)	62.49 (4)	<.001
Time × sex (factor + higher order factors)	15.36 (4)	.004
Time × age		
Factor + higher order factors	9.03 (4)	.06
Nonlinear	5.97 (2)	.05
Time × race (factor + higher order factors)	16.98 (10)	.08
Time × parental education (factor + higher order factors)	10.02 (6)	.12
Time × Material Deprivation Index		
Factor + higher order factors, percentile	9.45 (4)	.05
Nonlinear	5.65 (2)	.06
Time × Social Deprivation Index		
Factor + higher order factors, percentile	2.58 (4)	.63
Nonlinear	0.79 (2)	.67
Time × mechanism of injury (factor + higher order factors)	15.84 (10)	.10
Time × previous concussion maximum symptom duration (factor + higher order factors)	4.11 (2)	.13
Time × child history of migraine (factor + higher order factors)	8.18 (2)	.02
Time × HBI cognitive retrospective pre-injury		
Factor + higher order factors	10.36 (4)	.04
Nonlinear	2.37 (2)	.31
Time × HBI somatic retrospective preinjury		
Factor + higher order factors	5.00 (4)	.29
Nonlinear	2.01 (2)	.37
Time × group × sex (factor + higher order factors)	2.62 (2)	.27
**Overall (model-level) tests** ^b^		
Total nonlinear	26.35 (15)	.03
Total interaction	188.66 (57)	<.001
Total nonlinear + interaction	203.62 (62)	<.001
Total	795.03 (86)	<.001
Model log-likelihood ratio χ^2^ (*C* = 0.709)	707.57 (86)	<.001

^a^ If only 1 interaction is specified for a covariate, then its summary all interactions test will be the same as the test for that specific interaction.

^b^ Distribution of outcomes included in the ordinal model: 0 = 15; 1 = 86; 2 = 167; 3 = 201; 4 = 202; 5 = 244; 6 = 197; 7 = 203; 8 = 216; 9 = 209; 10 = 152; 11 = 107. The 743 unique participants contributed a total of 1999 rows of data to the model (each participant can contribute up to 3 rows, 1 per each study time point); 224 participants (23.2%) were excluded from model due to missing data; 120 of them (12.4%) do not have an outcome value across all time points.

Assessment of the partial effect plots (Figure) and postmodel fit contrasts (eTable 3 in Supplement 1) suggest group differences in optimal functioning were most notable at the earlier time points. At the 10-day follow-up, the concussion group had lower optimal functioning than the OI group among both females (OR, 0.24 [95% CI, 0.16-0.36]) and males (OR, 0.37 [95% CI, 0.26-0.53]). This difference persisted for females at 3 months (OR, 0.57 [95% CI, 0.35-0.93]), but was no longer present in males. Group differences were not significant for either sex at 6 months.

**Figure.**
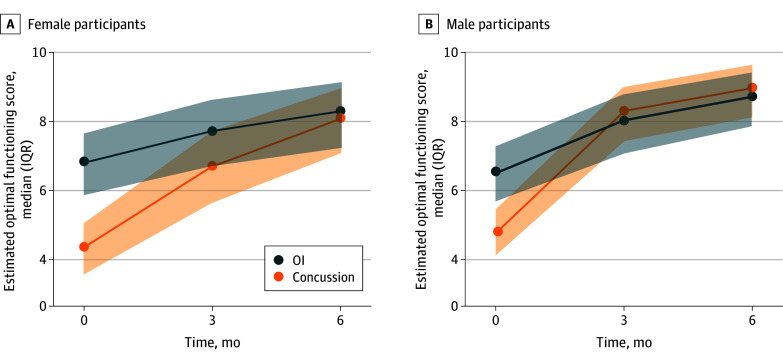
Partial Effect Plots From Multivariable Model Fit of the Associations Among Group, Time, and Sex With Optimal Functioning The model was adjusted to age 12.28 years, White race, parent with a Bachelor's degree, 28th percentile material deprivation index, 42nd percentile social deprivation index, mechanism of injury associated with a fall, less than 1 week symptom duration for previous concussion or no previous concussions, no history of migraine, 7 preinjury cognitive Health and Behavior Inventory items, and 1 preinjury somatic Health and Behavior Inventory item.

Optimal functioning increased over time in both groups and sexes. Across time points, optimal functioning increased in the OI group among both females (OR, 2.38 [95% CI, 1.36-4.16]) and males (OR, 3.65 [95% CI, 2.15-6.22]). Even larger increases in optimal functioning in the concussion group were apparent for both females (OR, 8.75 [95% CI, 4.98-15.39]) and males (OR, 11.58 [95% CI, 6.95-19.27]).

Sex differences were more consistently observed after concussion compared with OI. Males with concussion had greater odds of exhibiting higher optimal functioning than females, particularly at the 2 later time points (3 months: OR, 2.49 [95% CI, 1.67-3.73]; 6 months: OR, 1.75 [95% CI, 1.14-2.69]). For the OI group, the odds of males having greater optimal functioning than females were not significant at the 10-day (OR, 0.86 [95% CI, 0.57-1.31]), 3-month (OR, 1.19 [95% CI, 0.72-1.97]), or 6-month (OR, 1.33 [95% CI, 0.79-2.24]) follow-ups.

## Discussion

This cohort study found that fewer children who sustained concussion had optimal functioning compared with children who sustained OI, especially early in recovery. Group differences were apparent across domains, including motor-physical function, which might have been expected to be equal or worse after an OI. Optimal functioning became more likely with time since injury, but females who sustained concussion continued to have less optimal functioning 3 months after injury compared with males with concussion and with females with OI, whereas males with concussion did not differ from males with OI at 3 months. Six months after injury, the groups had comparable levels of optimal functioning.

As with studies focusing specifically on PCS, these findings can be interpreted with a reasonably optimistic view of concussion in which sequelae tend to be transient, with most children expected to make a full recovery in the long term. Previous reports suggest PCS resolve within 2 weeks^23^ with only one-third of participants reporting persisting symptoms after 1 month^6,24^ and no associations with intellectual functioning.^25^ However, the comprehensive and long-term approach in this study suggests that complete recovery for children, ie, they are considered and perceive themselves as functioning optimally across domains, may take well beyond the 1-month span typically cited for PCS resolution (and up to 6 months in females). While this more protracted course may seem surprising, it likely reflects our stringent operationalization of optimal functioning, requiring participants to meet high standards of performance and exhibit an absence of impairment. Such a conceptualization does not focus on obvious or overt symptom differences but instead may reflect more subtle variations that only emerge when considered across domains.

Females were less likely than males to meet criteria for optimal functioning even 6 months after injury. The presence and extent of sex differences after pediatric concussion continues to be debated, although most studies focus only on PCS. Ledoux et al^23^ found that girls were more symptomatic and had slower symptom change over 3 months after injury than boys. While female sex is associated with more baseline symptoms, sex does not appear to be a robust factor associated with recovery time in athletes.^26,27^ Methodological differences among studies may explain these discrepancies, with type of outcome and reporting method playing important roles. Poorer outcome in females is often interpreted in terms of self-reporting bias, such as females being more honest or more anxious. In the current study, sex differences were more apparent after concussion than OI, and were no longer apparent 6 months after injury, which may speak to sex differences in the biological effect of injury and not just psychological reporting bias. Other work from the same cohort supports this notion, showing a moderation between sex and brain structure and connectivity after pediatric concussion.^28,29^ Desai and colleagues^30^ also considered multiple domains (eg, return to school, return to noncontact sport, return to full sport, cognition, vision, vestibular) and found that females with pediatric concussion took longer to recover than males.

The definition of optimal functioning used in this study included measures of health-related quality of life, itself a multifaceted construct. A systematic review found that a small, but not negligible, subgroup experienced poor quality of life a year or more after pediatric concussion.^31^ Perceptions of reduced quality of life are often associated with sequelae in other areas, such as persisting symptoms,^2,4,32^ suggesting shared effects. Our definition also included indicators of positive functioning in the form of resilience and social support. Previous analyses from the same cohort concluded that psychological resilience is reduced after concussion and also affects recovery, and that greater resilience is associated with fewer PCS.^10^ Although resilience was used as an outcome variable in this study, preinjury resilience (eg, personal competence, tenacity, tolerance or negative affect, positive acceptance of change, control, and spiritual influences) also is associated with outcome after pediatric concussion.^9^ Overall, resilience has complex and dynamic associations with cognitive and brain reserve,^8^ which may be at play here, given the multifactorial definition of optimal outcome.

This study underscores a need to consider the heterogeneity of recovery trajectories depending on definitions of outcome, and the likelihood that more comprehensive operationalizations are likely to show a more protracted course. Mayer and colleagues^33^ used 10 distinct definitions of concussion recovery and found wide variability in recovery rates. Other work has focused on teasing out differences between physiological sequelae and clinical indicators, suggesting that the former may outlast symptom reports and a need to consider both.^34,35^ Haider et al^36^ conducted a systematic review of criteria used to define recovery and concluded that most studies use only symptom reports. Overall, our study aligns with repeated calls to consider the heterogeneity and complexity of pediatric concussion profiles and phenotypes, and need to take a comprehensive and holistic view to accurately determine outcome.^1,37,38^

### Limitations

This study has some limitations. First, we only considered biological sex, not gender, as a risk factor. Second, only self-report was used for questionnaires. While self-reported outcomes are advantageous in reflecting participants’ own recovery assessment, third-party and objective assessments may provide different insights. Third, the sample was recruited in large urban hospitals and may not represent the full spectrum of pediatric concussion. Likewise, the sample is relatively homogeneous and may not be generalizable to the population, although it is broadly representative in the Canadian context. Fourth, the indicators used to define optimal functioning were selected a posteriori based on those from the original protocol. Some variables did not have normative data, and cutoffs were based on the OI median score; however, participants in the OI group may also have exhibited less than optimal function. It was also not possible to set a normative threshold to calculate proportions of participants meeting optimal functioning. Thus, the score is meaningful when interpreted from a relative group perspective rather than an absolute one. Future work could seek to validate the score and determine reference levels for optimal functioning in the typically developing population. Furthermore, while the goal was specifically to examine positive outcomes, defining optimal functioning as requiring average or better functioning sets a high bar, and some participants may consider themselves to be fully recovered without reaching this stringent threshold.

## Conclusions

The findings of this cohort study suggest that children who sustain concussion may need 3 months or longer to be considered optimally recovered across physical, cognitive, socioemotional, and resilience domains. Clinically, these findings may help to explain why some children, especially girls, take longer to feel as though they are well even though subjective and objective assessments of symptoms or specific functions indicate absence of problems. Most children reach optimal functioning within 3 to 6 months; this information should be included in anticipatory guidance and reassurance to families.

## Notes

Supplement 1. **eTable 1.** Summary Statistics for the 11 Optimal Functioning Components and Score Criteria **eTable 2.** Comparison of Baseline Characteristics Between Participants Included and Excluded From Final Statistical Model **eTable 3.** Postmodel Fit Contrasts Quantifying the Relationship Among Group, Time, and Sex on Optimal Functioning **eFigure.** Distribution of Optimal Functioning Score and Subscores Over Time by Participant Group

Supplement 2.
The Pediatric Emergency Research Canada A-CAP Study Team

Supplement 3.
Data Sharing Statement
