# Supplementary material for: Optimal Recovery Following Pediatric Concussion
Source: JAMA Netw Open. 2025 Mar 19;8(3):e251092. doi: 10.1001/jamanetworkopen.2025.1092 (PMC11923687; doi:10.1001/jamanetworkopen.2025.1092)
Supplement: Supplement 1. — eTable 1. Summary Statistics for the 11 Optimal Functioning Components and Score Criteria eTable 2. Comparison of Baseline Characteristics Between Participants Included and Excluded From Final Statistical Model eTable 3. Postmodel Fit Contrasts Quantifying the Relationship Among Group, Time, and Sex on Optimal Functioning eFigure. Distribution of Optimal Functioning Score and Subscores Over Time by Participant Group [file jamanetwopen-e251092-s001.pdf]

Supplemental Online Content

Beauchamp MH, Tang K, Ledoux A, et al; Pediatric Emergency Research Canada A-CAP Study Team. Optimal recovery following pediatric concussion. *JAMA Netw Open*. 2025;8(3):e251092. doi:10.1001/jamanetworkopen.2025.1092

eTable 1. Summary Statistics for the 11 Optimal Functioning Components and Score Criteria

eTable 2. Comparison of Baseline Characteristics Between Participants Included and Excluded From Final Statistical Model

eTable 3. Postmodel Fit Contrasts Quantifying the Relationship Among Group, Time, and Sex on Optimal Functioning

eFigure. Distribution of Wellness Score and Subscores Over Time by Participant Group

This supplemental material has been provided by the authors to give readers additional information about their work.

© 2025 Beauchamp MH et al. *JAMA Network Open*.

eTable 1. Summary statistics for the 11 optimal functioning components and score criteria

				OI			Concussion			
#	Measure	criterion	*N*	*n*	median (Q1,Q3)	*n* (%) optimal	*n*	median (Q1,Q3)	*n* (%) optimal	*P*
1	**Total physical activity hours/week 10D**	≥7	813	275	6.8 (2.0, 15.8)	136 (49.5)	538	4.0 (1.2, 10.5)	200 (37.2)	<.001
2	**HBI somatic 10D**	≤2	829	278	2.0 (0.0, 5.0)	161 (57.9)	551	9.0 (5.0, 14.0)	60 (10.9)	<.001
3	**BESS tandem stance 10D**	≤3	773	236	2.0 (1.0, 3.0)	201 (85.2)	537	2.0 (1.0, 4.0)	375 (69.8)	<.001
4	**PedsQL physical activity 10D**	≥87.77	828	278	85.9 (71.9, 93.8)	122 (43.9)	550	81.2 (65.6, 90.6)	170 (30.9)	<.001
5	**CNS vital signs standard score 10D**	≥90	815	274	99.0 (87.0, 107.0)	193 (70.4)	541	93.0 (78.0, 105.0)	305 (56.4)	<.001
6	**HBI cognitive 10D**	≤4	829	278	4.0 (1.0, 9.0)	149 (53.6)	551	13.0 (6.0, 20.0)	102 (18.5)	<.001
7	**PedsQL school functioning 10D**	≥81.31	828	278	80.0 (65.0, 90.0)	104 (37.4)	550	70.0 (55.0, 80.0)	125 (22.7)	<.001
8	**PedsQL social functioning 10D**	≥84.97	828	278	90.0 (80.0, 100.0)	193 (69.4)	550	85.0 (70.0, 95.0)	317 (57.6)	0.001
9	**PedsQL emotional functioning 10D**	≥79.21	828	278	75.0 (60.0, 90.0)	138 (49.6)	550	70.0 (55.0, 85.0)	198 (36.0)	<.001
10	**CDRS total 10D**	≥30	829	278	30.0 (23.2, 34.0)	141 (50.7)	551	25.0 (20.0, 30.0)	149 (27.0)	<.001
11	**CASS total 10D**	≥214	820	276	214.0 (193.0, 241.0)	138 (50.0)	544	214.0 (192.0, 242.2)	274 (50.4)	0.92
1	**Total physical activity hours/week 3M**	≥7	721	248	14.6 (6.5, 26.5)	185 (74.6)	473	14.5 (6.5, 27.8)	350 (74.0)	0.86
2	**HBI somatic 3M**	≤2	728	248	2.0 (0.0, 5.0)	143 (57.7)	480	3.0 (0.0, 7.0)	225 (46.9)	0.006
3	**BESS tandem stance 3M**	≤3	710	240	1.5 (1.0, 3.0)	209 (87.1)	470	2.0 (1.0, 3.0)	381 (81.1)	0.04
4	**PedsQL physical activity 3M**	≥87.77	727	248	93.8 (84.4, 100.0)	165 (66.5)	479	93.8 (81.2, 100.0)	290 (60.5)	0.11
5	**CNS vital signs standard score 3M**	≥90	708	243	100.0 (89.5, 109.5)	182 (74.9)	465	96.0 (84.0, 107.0)	295 (63.4)	0.002
6	**HBI cognitive 3M**	≤4	728	248	3.0 (0.0, 11.0)	136 (54.8)	480	5.0 (0.0, 13.0)	227 (47.3)	0.05
7	**PedsQL school functioning 3M**	≥81.31	727	248	85.0 (70.0, 95.0)	136 (54.8)	479	80.0 (65.0, 95.0)	218 (45.5)	0.02
8	**PedsQL social functioning 3M**	≥84.97	727	248	95.0 (80.0, 100.0)	184 (74.2)	479	90.0 (80.0, 100.0)	331 (69.1)	0.15
9	**PedsQL emotional functioning 3M**	≥79.21	727	248	80.0 (65.0, 95.0)	135 (54.4)	479	75.0 (60.0, 90.0)	232 (48.4)	0.13
10	**CDRS total 3M**	≥30	727	248	29.0 (23.8, 34.0)	115 (46.4)	479	27.0 (21.0, 33.0)	190 (39.7)	0.08
11	**CASS total 3M**	≥214	724	248	221.0 (194.0, 243.2)	142 (57.3)	476	220.0 (188.0, 245.0)	271 (56.9)	0.93
1	**Total physical activity hours/week 6M**	≥7	693	237	14.8 (7.0, 28.0)	179 (75.5)	456	16.4 (7.5, 30.0)	351 (77.0)	0.67
2	**HBI somatic 6M**	≤2	701	239	2.0 (0.0, 5.0)	134 (56.1)	462	3.0 (0.0, 7.0)	227 (49.1)	0.08
3	**BESS tandem stance 6M**	≤3	688	237	1.0 (0.0, 3.0)	204 (86.1)	451	1.0 (0.0, 3.0)	382 (84.7)	0.63
4	**PedsQL physical activity 6M**	≥87.77	699	238	93.8 (81.2, 100.0)	153 (64.3)	461	93.8 (87.5, 100.0)	320 (69.4)	0.17
5	**CNS vital signs standard score 6M**	≥90	690	238	101.5 (91.0, 111.0)	188 (79.0)	452	98.0 (86.0, 108.0)	313 (69.2)	0.006
6	**HBI cognitive 6M**	≤4	701	239	4.0 (0.0, 11.0)	121 (50.6)	462	5.0 (0.0, 13.0)	216 (46.8)	0.33

© 2025 Beauchamp MH et al. *JAMA Network Open*.

#	Measure	criterion	*N*	OI *n*	OI median (Q1,Q3)	OI *n* (%) optimal	Concussion *n*	Concussion median (Q1,Q3)	Concussion *n* (%) optimal	*P*
7	**PedsQL school functioning 6M**	≥81.31	699	238	85.0 (70.0, 95.0)	131 (55.0)	461	85.0 (65.0, 95.0)	234 (50.8)	0.28
8	**PedsQL social functioning 6M**	≥84.97	699	238	90.0 (80.0, 100.0)	172 (72.3)	461	95.0 (80.0, 100.0)	324 (70.3)	0.58
9	**PedsQL emotional functioning 6M**	≥79.21	699	238	80.0 (65.0, 95.0)	125 (52.5)	461	75.0 (60.0, 95.0)	228 (49.5)	0.44
10	**CDRS total 6M**	≥30	700	238	30.0 (24.0, 35.0)	126 (52.9)	462	27.0 (22.0, 34.0)	186 (40.3)	0.001
11	**CASS total 6M**	≥214	695	238	222.0 (192.0, 246.0)	139 (58.4)	457	223.0 (193.0, 249.0)	263 (57.5)	0.83

P values derived from Pearson chi-square tests comparing % optimal between patient groups

Abbreviations: BESS: Balance Error Scoring System; C: Child, CASS: Child and Adolescent Support Scale, CDRS: Connor-Davidson Resilience Scale, CNS Vital Signs: Central Nervous System Vital Signs, HBI: Health and Behavior Inventory, HLBQ: Healthy Lifestyle Behaviours Questionnaire, NA: Not Applicable, PedsQL: Pediatric Quality of Life Inventory

© 2025 Beauchamp MH et al. *JAMA Network Open*.

eTable 2. Comparison of baseline characteristics between participants included and excluded from final statistical model

Variable	*N*	Included (*n*=743)		Excluded (*n*=224)		*P*
		n	Value	*n*	Value	
**Group**, *n* (%)	967	743		224		0.06
OI			245 (33.0)		89 (39.7)	
Concussion			498 (67.0)		135 (60.3)	
**Site**, *n* (%)	967	743		224		0.65
Calgary			178 (24.0)		48 (21.4)	
Edmonton			146 (19.7)		38 (17.0)	
Montreal			77 (10.4)		23 (10.3)	
Ottawa			165 (22.2)		52 (23.2)	
Vancouver			177 (23.8)		63 (28.1)	
**Age** *years*, median (IQR)	967	743	12.3 (10.5, 14.4)	224	12.3 (10.5, 14.1)	0.69
**Sex**, *n* (%)	967	743		224		0.37
Female			317 (42.7)		88 (39.3)	
Male			426 (57.3)		136 (60.7)	
**Race/Ethnicity***, *n* (%)	834	743		91		0.02
Asian			61 (8.2)		14 (15.4)	
Black			25 (3.4)		3 (3.3)	
Hispanic			24 (3.2)		1 (1.1)	
Indigenous			13 (1.7)		3 (3.3)	
White			531 (71.5)		52 (57.1)	
Other / Multi-racial			89 (12.0)		18 (19.8)	
**Parental education**, *n* (%)	824	743		81		0.51
High school or less			117 (15.7)		12 (14.8)	
Trades / 2-year college			220 (29.6)		27 (33.3)	
Bachelor’s degree			275 (37.0)		24 (29.6)	
Higher than Bachelor’s degree			131 (17.6)		18 (22.2)	
**Material deprivation index (percentile)**, median (IQR)	918	743	26.0 (11.0, 53.0)	175	35.0 (12.0, 59.0)	0.10
**Social deprivation index (percentile)**, median (IQR)	918	743	41.0 (23.0, 66.0)	175	46.0 (22.0, 64.5)	0.75
**Mechanism of injury**, *n* (%)	867	743		124		0.36
Bicycle related			19 (2.6)		2 (1.6)	
Fall			356 (47.9)		68 (54.8)	

© 2025 Beauchamp MH et al. *JAMA Network Open*.

Variable	*N*	Included (*n*=743)		Excluded (*n*=224)		*P*
		n	**Value**	***n***	**Value**	
Motor vehicle collision			8 (1.1)		2 (1.6)	
Struck object			216 (29.1)		31 (25.0)	
Struck person			118 (15.9)		20 (16.1)	
Other			26 (3.5)		1 (0.8)	
**Previous concussion maximum symptom duration**, *n* (%)	947	743		204		0.95
<1 week / no previous concussions			606 (81.6)		166 (81.4)	
1+ week(s)			137 (18.4)		38 (18.6)	
**Child history of migraine**, *n* (%)	945	743	47 (6.3)	202	10 (5.0)	0.47
**HBI cognitive retrospective pre-injury**, median (IQR)	836	743	7.0 (1.0, 14.0)	93	8.0 (3.0, 15.0)	0.31
**HBI somatic retrospective pre-injury**, median (IQR)	836	743	1.0 (0.0, 4.0)	93	2.0 (0.0, 5.0)	0.03

Notes: included = contributed at least 1 row of data to final model (i.e. if at least 1 of 3 study time-points had complete data); *P* value from Wilcoxon test (continuous) or Pearson chi-square test (categorical); HBI = Health and Behavior Inventory; *Race/Ethnicity were reported by the participant's primary caregiver on an in-house socio-demographic questionnaire. The "Other / Multi-racial" category was an original survey option that respondents could self-select.

© 2025 Beauchamp MH et al. *JAMA Network Open*.

eTable 3. Post model fit contrasts quantifying the relationship among group, time, and sex on optimal functioning

Contrast	Interacting Factors	OR (95%CI)
**Group: Concussion vs OI**	Sex=Female, Time=PA	0.24 (0.16, 0.36)
**Group: Concussion vs OI**	Sex=Female, Time=3M	0.57 (0.35, 0.93)
**Group: Concussion vs OI**	Sex=Female, Time=6M	0.89 (0.55, 1.45)
**Group: Concussion vs OI**	Sex=Male, Time=PA	0.37 (0.26, 0.53)
**Group: Concussion vs OI**	Sex=Male, Time=3M	1.20 (0.78, 1.82)
**Group: Concussion vs OI**	Sex=Male, Time=6M	1.18 (0.76, 1.83)
**Time: 3M vs 10D**	Sex=Female, Group=OI	1.69 (1.02, 2.83)
**Time: 6M vs 10D**	Sex=Female, Group=OI	2.38 (1.36, 4.16)
**Time: 6M vs 3M**	Sex=Female, Group=OI	1.40 (0.85, 2.32)
**Time: 3M vs 10D**	Sex=Female, Group=Concussion	3.97 (2.39, 6.60)
**Time: 6M vs 10D**	Sex=Female, Group=Concussion	8.75 (4.98, 15.39)
**Time: 6M vs 3M**	Sex=Female, Group=Concussion	2.20 (1.42, 3.41)
**Time: 3M vs 10D**	Sex=Male, Group=OI	2.33 (1.42, 3.80)
**Time: 6M vs 10D**	Sex=Male, Group=OI	3.65 (2.15, 6.22)
**Time: 6M vs 3M**	Sex=Male, Group=OI	1.57 (1.01, 2.45)
**Time: 3M vs 10D**	Sex=Male, Group=Concussion	7.47 (4.69, 11.90)
**Time: 6M vs 10D**	Sex=Male, Group=Concussion	11.58 (6.95, 19.27)
**Time: 6M vs 3M**	Sex=Male, Group=Concussion	1.55 (0.99, 2.43)
**Sex: Male vs Female**	Group=OI, Time=10D	0.86 (0.57, 1.31)
**Sex: Male vs Female**	Group=OI, Time=3M	1.19 (0.72, 1.97)
**Sex: Male vs Female**	Group=OI, Time=6M	1.33 (0.79, 2.24)
**Sex: Male vs Female**	Group=Concussion, Time=10D	1.33 (0.97, 1.82)
**Sex: Male vs Female**	Group=Concussion, Time=3M	2.49 (1.67, 3.73)
**Sex: Male vs Female**	Group=Concussion, Time=6M	1.75 (1.14, 2.69)

Note: OR, adjusted odds ratio; 95%CI, 95% confidence intervals. Effect sizes for all relevant interacting factor combinations from multivariable model fit are presented. The study timepoints were post-acute (PA), 3-months post-injury (3M), and 6-months post-injury (6M). OI= orthopedic injury

© 2025 Beauchamp MH et al. *JAMA Network Open*.

eFigure 1. Distribution of optimal functioning score and subscores over time by participant group

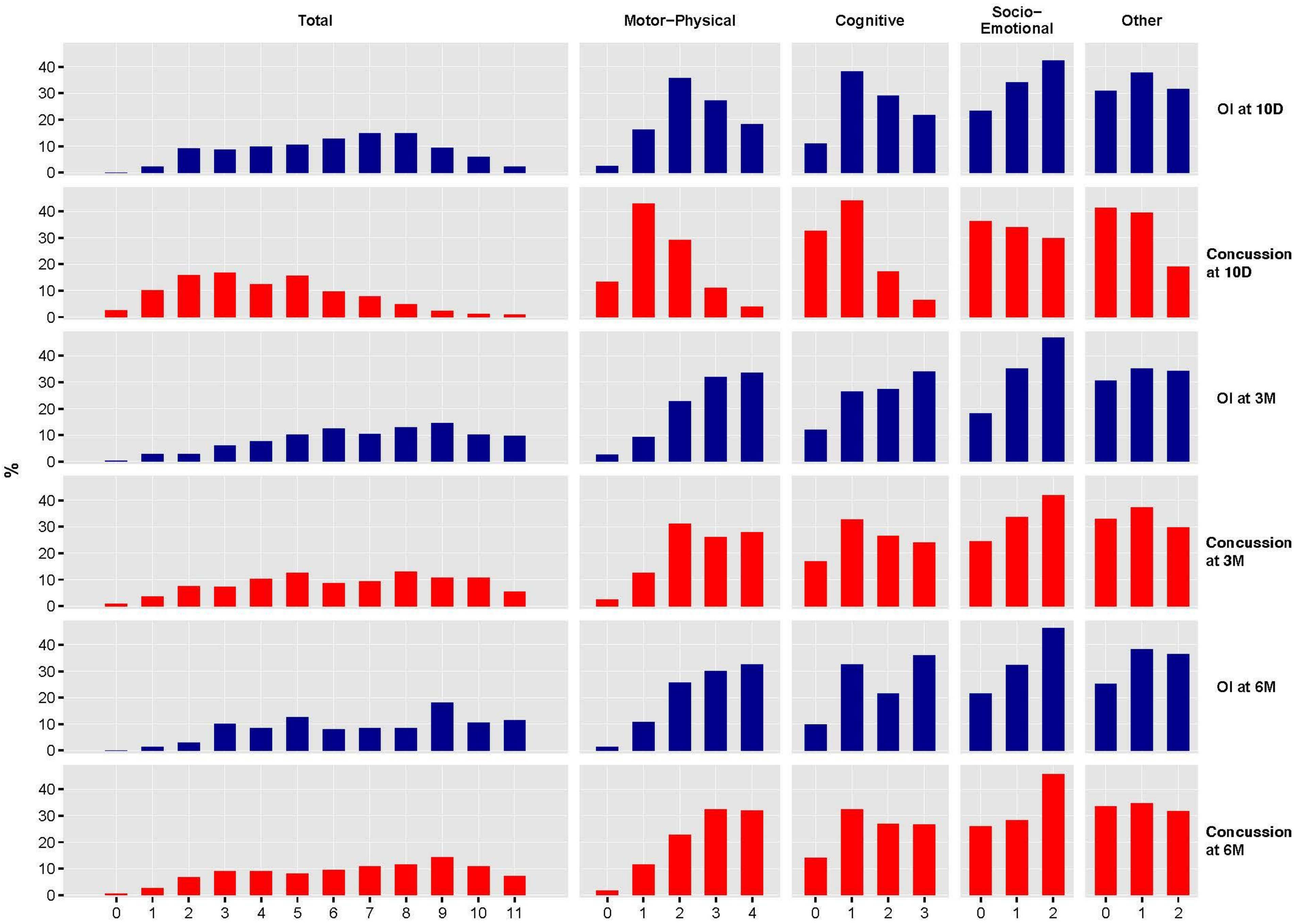

© 2025 Beauchamp MH et al. *JAMA Network Open*.
