# Supplementary material for: Optimal Recovery Following Pediatric Concussion
Source: JAMA Netw Open. 2025 Mar 19;8(3):e251092. doi: 10.1001/jamanetworkopen.2025.1092 (PMC11923687; doi:10.1001/jamanetworkopen.2025.1092)
Supplement: Supplement 2. — The Pediatric Emergency Research Canada A-CAP Study Team [file jamanetwopen-e251092-s002.pdf]

Supplemental Online Content: Nonauthor Collaborators
*First name, last name, and suffix (if applicable) are required and will appear in PubMed.

*Group Name(s): Pediatric Emergency Research Canada ACAP Study Team							
***First Name and Middle Initial(s)**	***Last Name**	***Suffix (eg, Jr, III)**	Academic Degrees	Institution	Location (city, state/province, country)	Role or Contribution, eg, chair, principal investigator	Group (if more than 1 Group listed in the byline) and/or Subgroup (eg, Steering Committee)
Bruce H	Bjornson		MD	BC Children's Hospital	Vancouver, British Columbia, Canada	co-site principal investigator	
Jocelyn	Gravel		MD	University of Montreal & CHU Sainte-Justine Azrieli Research Center	Montreal, Quebec, Canada	co-site principal investigator	
Angelo	Mikrogianakis		MD	McMaster University & McMaster Children's Hospital	Hamilton, Ontario, Canada	co-investigator	
Bradley	Goodyear		PhD	University of Calgary	Calgary, Alberta, Canada	co-investigator	
Nishard	Abdeen		MD	University of Ottawa	Ottawa, Ontario, Canada	collaborator	
Christian	Beaulieu		PhD	University of Alberta	Calgary, Alberta, Canada	co-investigator	
Mathieu	Dehaes		PhD	University of Montreal & CHU Sainte-Justine Azrieli Research Center	Montreal, Quebec, Canada	collaborator	
Sylvain	Deschenes		PhD	CHU Sainte-Justine	Montreal, Quebec, Canada	collaborator	
Catherine	Lebel		PhD	University of Calgary & Alberta Children's Hospital	Calgary, Alberta, Canada	co-investigator	
Ryan	Lamont		PhD	University of Calgary	Calgary, Alberta, Canada	co-investigator	
Tyler	Williamson		PhD	University of Calgary	Calgary, Alberta, Canada	collaborator	
Karen M	Barlow		MD	University of Queensland	Brisbane, Queensland, Australia	collaborator	
Brian L	Brooks		PhD	Alberta Children's Hospital	Calgary, Alberta, Canada	co-investigator	
Carolyn	Emery		PhD	University of Calgary	Calgary, Alberta, Canada	co-investigator	
Stephen B	Freedman		MD	Alberta Children's Hospital	Calgary, Alberta, Canada	co-investigator	
Lianne	Tomfohr-Madsen		PhD	BC Children's Hospital	Vancouver, British Columbia, Canada	co-investigator	
Kelly	Mrklas			Alberta Health Services	Calgary, Alberta, Canada	collaborator	
Kathryn J	Schneider		PhD	University of Calgary	Calgary, Alberta, Canada	co-investigator	
