# Supplementary material for: Optimal Recovery Following Pediatric Concussion
Source: JAMA Netw Open. 2025 Mar 19;8(3):e251092. doi: 10.1001/jamanetworkopen.2025.1092 (PMC11923687; doi:10.1001/jamanetworkopen.2025.1092)
Supplement: Supplement 3. — Data Sharing Statement [file jamanetwopen-e251092-s003.pdf]

Data Sharing Statement

Data

Data available: Yes
Data types: Deidentified participant data
How to access data: A dataset with deidentified participant data and a data dictionary will be made available upon reasonable request from any qualified investigator, subject to a signed data access agreement.
When available: With publication

Supporting Documents

Document types: None

Additional Information

Who can access the data: Qualified investigators subject to a signed data access agreement.
Types of analyses: Any purpose
Mechanisms of data availability: After approval of a signed data access agreement.
